# The impact of the let-7 family on the pathophysiological mechanisms of traumatic brain injury: a systematic review

**DOI:** 10.3389/fneur.2025.1667381

**Published:** 2025-10-02

**Authors:** Natalia Radenza, Renata Mangione, Saviana Antonella Barbati, Francesco Bellia, Giuseppe Caruso, Antonio Belli, Giuseppe Lazzarino, Barbara Tavazzi, Giacomo Lazzarino, Angela Maria Amorini, Valentina Di Pietro

**Affiliations:** ^1^Department of Biomedical and Biotechnological Sciences, University of Catania, Catania, Italy; ^2^Departmental Faculty of Medicine, UniCamillus-Saint Camillus International University of Health and Medical Sciences, Rome, Italy; ^3^IRCCS San Camillo Hospital, Venice, Italy; ^4^Neuroscience and Ophthalmology, Department of Inflammation and Ageing, School of Infection, Inflammation and Immunology, College of Medicine and Health, University of Birmingham, Birmingham, United Kingdom; ^5^LTA-Biotech srl, Paternò, CT, Italy; ^6^Birmingham Centre for Neurogenetics, Birmingham, United Kingdom

**Keywords:** microRNA, let-7, traumatic brain injury, biomarkers, biofluids, nervous cell metabolism

## Abstract

**Background:**

The lethal-7 (let-7) family of microRNAs (miRNAs) plays a crucial role in regulating key biological functions, including cell differentiation, inflammation, metabolism, and proliferation. Their dysregulation has been implicated in various diseases, including cancer and neurological disorders. Despite extensive knowledge of their roles in normal physiology and disease, the involvement of let-7 miRNAs in the pathophysiology of traumatic brain injury (TBI) remains incompletely understood.

**Objective:**

To systematically identify and analyze the differential expression of let-7 family members following TBI across human and animal models and to assess their potential role as diagnostic biomarkers and therapeutic targets.

**Methods:**

A systematic review was conducted in accordance with Preferred Reporting Items for Systematic Reviews and Meta-Analyses (PRISMA) guidelines. Literature was retrieved from PubMed, EMBASE, and Web of Science using predefined keywords based on a PICO framework. Studies were included if they reported on let-7 family expression post-TBI in humans or animals. Risk of bias was assessed using the Evidence Project and SYRCLE tools. Data were extracted regarding species, sample type, TBI model, time points, and let-7 expression profiles. PROSPERO 2025 CRD420251129282. Available from https://www.crd.york.ac.uk/PROSPERO/view/CRD420251129282.

**Results:**

Out of 41 initially identified records, 15 studies met the inclusion criteria. In human studies, upregulation of let-7 family members (e.g., let-7a, b, c, f, i) was consistently observed in peripheral biofluids (serum, plasma, saliva) following mild to severe TBI. However, cerebrospinal fluid (CSF) levels showed mixed or decreased expression patterns. In contrast, animal studies have shown predominant downregulation of let-7 in brain tissues post-TBI, with some evidence suggesting their role in modulating neuroinflammatory responses, apoptosis, and energy metabolism. Let-7c and let-7i were particularly implicated in the modulation of microglial activation, IL-6 regulation, and STING signaling pathways. Limited mechanistic data suggest let7′s involvement in glucose metabolism, N-acetylaspartate homeostasis, and antioxidant response.

**Conclusions:**

The let-7 family exhibits divergent expression trends in tissue and biofluids following TBI, highlighting their potential as non-invasive biomarkers. Their regulatory roles in inflammation, metabolism, and neuroprotection suggest therapeutic promise. However, current evidence remains fragmented, and further mechanistic studies are necessary to validate their function in post-TBI recovery and to explore their utility as clinical biomarkers or treatment targets.

## 1 Introduction

The lethal-7 (let-7) microRNA (miRNA, miR), named because its knockout was lethal during development (1), was the first miRNA discovered in humans (2). It is well conserved across species (3), from *Caenorhabditis elegans* (*C. elegans*) to humans, even though higher animals tend to have increased numbers of let-7 isoforms. Both *C. elegans* and *Drosophila melanogaster* (*D. melanogaster*) possess a singular let-7 isoform (let-7a), while humans and mice both possess 10 isoforms (miR-98, miR-202, and let-7a, 7b, 7c, 7d, 7e, 7f, 7g, and 7i) across 12 and 3 genetic loci, respectively (4, 5). The let-7 isoforms (apart from miR-202) contain a conserved ‘seed sequence' with the nucleotides GAGGUAG in positions 2 to 8 of each miRNA molecule (5, 6). The preservation of this sequence between isoforms implicates similarly conserved biological targets and evolutionarily retained functions.

Expression of let-7 is ubiquitous in adult mammalian tissues and known to increase throughout development (7), but the specific expression levels of each let-7 isoform are context-dependent and tightly regulated (8). Due to its roles in developmental differentiation, proliferation, inflammation, and cellular metabolism, dysregulation of let-7 expression has been implicated in several pathologies, including cancers and cerebral and cardiovascular disorders (5).

In embryogenesis, let-7 plays a crucial role in controlling the development from the first trimester to birth (9–11). In the central nervous system (CNS), let-7 enhances glycogen biosynthesis by suppressing HMGA2 and NOTCH (12) and is implicated in maintaining endothelial cell integrity (3), thus providing an optimal functionality of the blood-brain barrier (13). Given its role as a regulator of angiogenesis, let-7 can also control cancer progression (14). Reduced let-7 expression is a common hallmark of most cancers (15), leading to the upregulation of genes critical for DNA replication, such as programmed cell death ligand 1 (PD-L1) (16) and high mobility group AT-hook 2 (HMGA2) (17). Additionally, apoptotic genes like caspase 3 (18), caspase 8, and B-cell CLL/lymphoma (BCL) (19), or the pluripotency transcription factors NANOG, SOX2, SOX9, OCT4, KLF4, and c-Myc are modulated by let-7 and help the survival of cancer stem cells (CSCs) (20, 21).

The let-7 family appears critical also for the progression of inflammation, with various members associated with early inflammatory pathways (5) through the well-established mechanism of action via the LIN28/let-7 axis. LIN28B has been identified as a molecular link between inflammation and cancer through a positive feedback loop involving NF-κB activation and the release of repression from the let-7-regulated IL-6 gene (22). More recent studies have associated the LIN28/let-7 axis with glucose metabolism because of its regulatory effects on PDK1, the function of which is to inhibit the activity of the pyruvate dehydrogenase (PDH) subunits of the pyruvate dehydrogenase complex (PDHC). Increased glucose metabolism is attributed to LIN28A/B-mediated repression of let-7 (23). In addition, in breast cancer, whilst LIN28A and LIN28B promote the oxidative metabolism of glucose, let-7, by acting on PDK1, promotes glycolytic consumption of glucose in a hypoxia-independent manner. This study suggests a direct regulation of the LIN28/let-7 axis in the glycolytic/oxidative modulation of glucose metabolism, even under normal oxygen levels (24). Inhibition of the enzymatic function of PDH shifts glucose-dependent energy production from mitochondrial oxidative phosphorylation (OXPHOS) to glycolysis, thus acting as a gatekeeper of glucose metabolism. Increased activity in breast cancer stem cells (BCSCs) is also associated with elevated expression of the long non-coding RNA H19, which sequesters let-7 miRNA, leading to activation of HIF1a and enhancing downstream target PDK1 expression (25).

**Figure 1:**
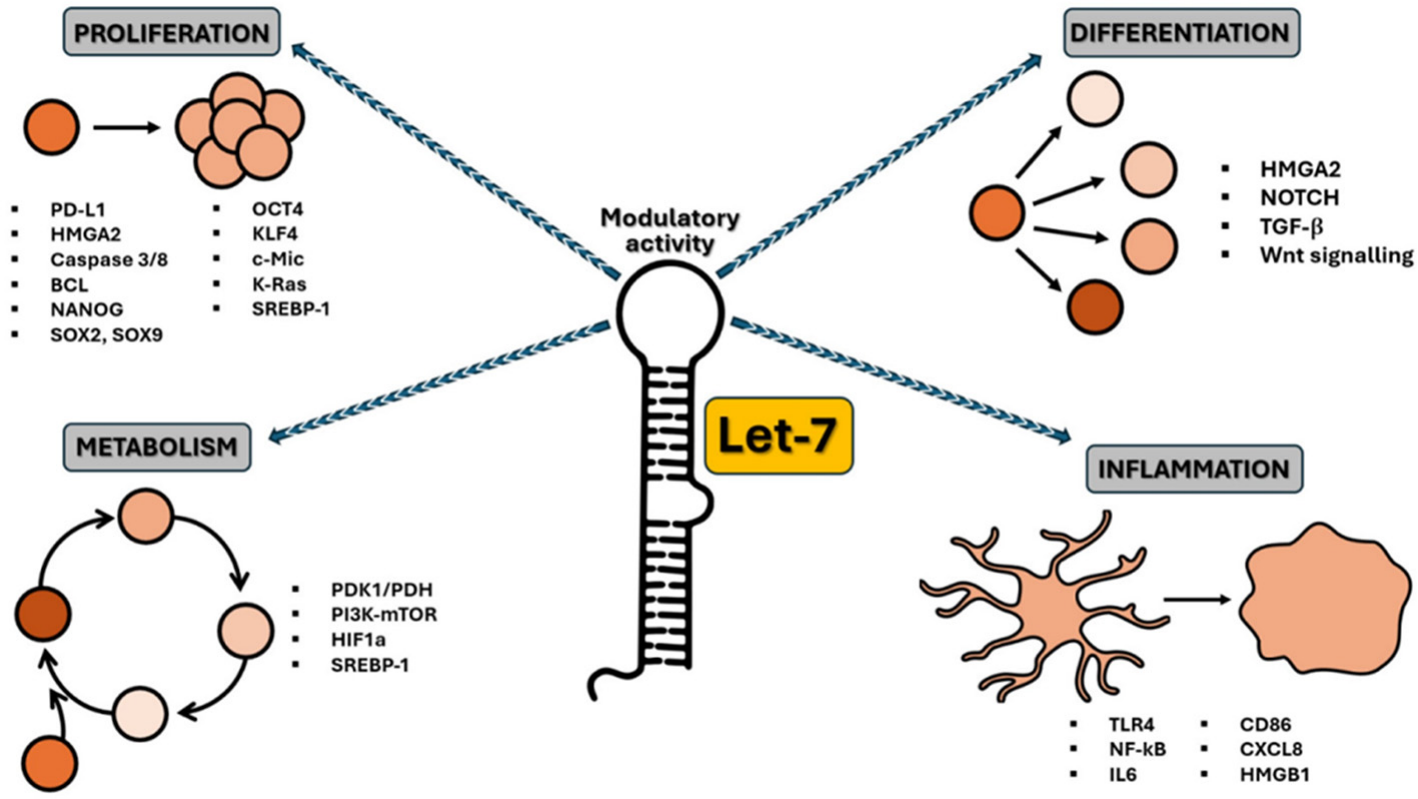
Schematic representation of the let-7 family activity as a crucial regulator of various cellular processes. Let-7 miRNAs regulate the cell cycle, particularly by inhibiting genes that drive cell division, thereby preventing uncontrolled proliferation. Let-7 can also act as a tumor suppressor, inhibiting the expression of oncogenes such as RAS, MYC, and HMGA2 and helping to prevent cancer progression. During development, let-7 miRNAs control cell differentiation by promoting the transition from stem cells to differentiated cells. Let-7 is also involved in regulating metabolic pathways, influencing lipid and glucose metabolism, and maintaining cell energy homeostasis. In the immune system, let-7 miRNAs modulate inflammation and immune cell activation, contributing to immune response regulation.

Furthermore, elevated expression of LIN28A/B enhances *de novo* fatty acid synthesis, promoting cancer progression via SREBP1. Lin28A/B directly associates with the mRNAs of both SREBP-1 and SCAP, enhancing the translation and maturation of SREBP-1, thereby accelerating metabolic conversion of saturated and unsaturated fatty acids and protecting cancer cells from lipotoxicity (26). A summary of the aforementioned functions of the let-7 family is presented in Table 1 and illustrated in Figure 1.

**Table 1:** Summary of current knowledge of the functional roles of the let-7 family.

**Function**	**Key finding**	**Reference**
Development and cell differentiation	Let-7 family identified as a heterochronic gene regulating specific proteins to coordinate developmental timing in *C. elegans*	(1, 2)
	Developmental stage in *C. elegans, D. Melanogaster*, chickens, and mice. In mammals, let-7 expression is high during embryogenesis and brain development. Cluster1-a, cluster1-b, cluster1-c are involved in the mediation of hematopoietic stem cells and progenitor cells homeostasis, differentiation, and phenotypic cell fate, through the respective suppression and activation of TGF-β and Wnt signaling pathways	(8)
	Let-7 performed an essential role in controlling cellular proliferation in the blastocyst and subsequent tissue development	(3)
	Let-7 miRNAs play a functional role in the development of human neural progenitors by suppressing HMGA2 and NOTCH to regulate glycogenesis. Its normal expression is also considered critical for the maintenance of the blood–brain barrier	(12)
	Let-7 expression throughout the development and maintenance of the cardiovascular system is highly significant in the protection and recovery of both endothelial cell and BBB integrity during normal conditions, as well as times of stress	(5)
Proliferation and tumor suppressor	Let-7 has been found to modulate signaling between fibroblast growth factors (FGF) and TGF-β in endothelial cells, limiting endothelial-to-mesenchymal transition and consequent proliferation	(81)
	Let-7 suppresses the translation of several oncogenes, including Myc, K-Ras, and HMGA2. Let-7 is deregulated in a wide variety of cancer types and influences CSC maintenance, metabolism, tumorigenesis, and metastasis. Let-7g was demonstrated to inhibit the expression of RAS in non-small cell lung cancer and consequently arrest tumorigenesis	(5, 8, 82–85)
	Increased let-7a expression represses chemoresistance and tumorigenicity through the ablation of CSC-like properties. In gastric cancer, let-7b was shown to directly suppress c-myc expression to promote gastric stem cell differentiation and drug sensitivity	(86, 87)
	The biogenesis of let-7 is inhibited by LIN28A and LIN28B, two highly related RBPs and proto-oncogenes. Activation of either is responsible for the global post-transcriptional downregulation of let-7 miRNAs in many cancers, which is associated with advanced tumor stages	(82)
	Wnt/β-catenin pathway represses let-7 expression post-transcriptionally by the transactivation of LIN28B to augment breast CSC phenotypes	(66)
	LIN28A facilitates glioblastoma neurosphere formation through constitutively activating K-Ras and suppressing let-7b and let-7. Targeting LIN28 and/or restoring let-7 expression, by inhibiting tumor metabolic activities in CSCs, may provide potential therapeutic strategies for cancer treatment	(88)
	Dysregulation of let-7 is thought to be influential in the development of vascular collaterals that support tumors	(89)
	Elevated LIN28A/B expression boosts fatty acid synthesis, aiding cancer progression through SREBP1. LIN28A/B interact with SREBP-1 and SCAP mRNAs, increasing SREBP-1 translation and maturation, speeding up the conversion of fatty acids, and protecting cancer cells from lipotoxicity	(26)
Immune response	Let-7g can improve several endothelial functions, including a decrease in senescence, inflammation, monocyte adhesion, and an increase in angiogenesis	(90)
	Let-7c regulates dental inflammation by negatively affecting the DMP1-mediated NF-κB signaling pathway	(91)
	Let-7i is involved in leukocyte modulation by inhibiting CD86, CXCL8, and HMGB1 expression	(92)
	Let-7 family members differentially act to influence microglial functions	(93)
	Let-7a targets HMGB1, which mediates microglial activation	(94)
	Let-7 and miR-98 resulted in reduced leukocyte adhesion to and migration across endothelium, diminished expression of pro-inflammatory cytokines, and increased BBB tightness	(95)
	Let-7i downregulates TLR4 and MMP9 in order to reduce pro-inflammatory signaling and protect BBB integrity	(13)
	Macrophages exhibit high expression levels of miRNA; let-7c inhibits the polarization of macrophages toward the M1 phenotype while enhancing M2 polarization, modulating macrophage plasticity	(74)
	Let-7 directly inhibits IL6 expression	(96)
	Let-7 can also target receptor TLR4, affecting the inflammatory response	(97)
	Let-7 is suppressed by LIN28 transcription induced by NFkB	(22)
Metabolism	PDK1 blocks glucose from entering mitochondrial oxidative phosphorylation (OXPHOS) and promotes aerobic glycolysis. In breast cancer stem cells (BCSCs), increased activity involves the long non-coding RNA H19 sequestering let-7 miRNA, which activates HIF1a and raises PDK1 expression	(24)
	Early studies with transgenic mice showed that the let-7/LIN28 axis regulates glucose metabolism. LIN28A/B represses let-7, which enhances glucose metabolism by activating the insulin-P13K-mTOR pathway and increasing insulin sensitivity	(25)
Neuroprotection and Neurodegeneration	Higher levels of let-7g^*^ and miR-98 expression have been associated with decreased neurotoxicity following stroke and improved neurovascular perfusion	(98)
	Dying neurons release let-7, which acts as a damage-associated molecular pattern (DAMP) by binding to the TLR7 receptor. This interaction activates microglia and macrophages, leading to increased neurotoxicity.	(94)
	In EAE, let-7 worsens disease by promoting harmful immune cell differentiation	(99)
	In newts, reducing let-7 is essential for tail regeneration, as let-7 mimics hinder this process	(100)
	Increased levels of let-7 have been suggested as potential biomarkers for multiple sclerosis, stroke, and Alzheimer's disease	(101)
	miR-98 downregulates endothelial tight junction proteins, negatively affecting BBB integrity	(102)
	miR-98 protects BBB integrity to positively influence ischemic stroke outcomes	(103)
	miR-let-7a suppresses α-Synuclein-induced microglia inflammation through targeting STAT3 in Parkinson's disease	(104)

Despite the numerous evidence of let-7 implication in regulating the biological and metabolic functioning of nervous cells, there are no direct studies in models of neurodegeneration that have allowed us to establish whether its roles are neuroprotective or neurodegenerative. Most existing data link altered let-7 expression to neurological disorders such as multiple sclerosis, stroke, and Alzheimer's disease (27), while its role in traumatic brain injury (TBI) remains understudied. TBI is a leading cause of disability worldwide and the most frequent cause of death in individuals under 35 in Western countries (28).

Its global incidence is estimated at over 900 cases per 100,000 people annually, corresponding to nearly 69 million new cases each year. By 2030, the burden of TBI-related disability is expected to surpass that of Alzheimer's disease and cerebrovascular disorders, creating significant personal and socioeconomic costs (29, 30).

TBI is defined as a non-congenital injury to the brain caused by the nervous cell absorption of part of the external physical forces acting at the time of injury (primary insult), which induces the sudden alteration of a significant number of biochemical, metabolic, and molecular processes (secondary insult), modifying brain cell homeostasis and provoking transient or long-lasting (up to permanent) impairment of consciousness, neurocognitive deficits, neuromotor disabilities, or psychological disturbances. Grossly, TBIs are classified using the Glasgow Coma Scale (GCS), which categorizes TBI patients into mild (GCS range 13–15), moderate (GCS range 9–12), and severe (GCS range 3–8), based on a score obtained after specific neurological assessments, such as eye opening, motor, and verbal responses.

Despite this alarming epidemiological evidence, effective treatments remain scarce, and researchers are just beginning to unravel the complex cellular, molecular, and biochemical changes that influence TBI outcomes (31–35). It is now well accepted that the concept of the neurometabolic cascade involves a change in ionic cell homeostasis, mitochondrial dysfunction with energy penalty, sustained oxidative/nitrosative stress, glutamate excitotoxicity, glucose dysmetabolism, profound changes in protein and gene expressions, dangerous neuroinflammation, damage to the BBB permeability, and induction of apoptosis with a consequent significant increase in the nervous cell mortality rate (36, 37). The duration and the severity of the aforementioned neurochemical alterations are strictly dependent on the amount of energy absorbed at the time of impact and, ultimately, on the biomechanics of TBI.

Patients with mild (mTBI) injuries (including concussions, a peculiar type of mTBI frequently encountered in sports) often go undiagnosed because they are frequently characterized by rapidly resolving clinical symptoms. Notwithstanding, experimental and clinical studies have shown that mTBI and concussions provoke biochemical/metabolic/molecular changes in nervous cells lasting much longer than symptom resolution, which may have severe pathological and neurological consequences, particularly in the case of repeated injuries (38).

Diagnostic approaches for TBI rely on imaging and biomarkers. Although computed tomography (CT) remains the preferred method for assessing focal injuries, it lacks the sensitivity to effectively detect diffuse damage (39). Magnetic resonance imaging (MRI) offers greater resolution but is costly and less accessible (40). Among biochemical markers, proteins such as S100b, GFAP, and ubiquitin C-terminal hydrolase-1 (UCH-L1) (41, 42) have been widely studied, and the combination of GFAP and UCH-L1 has recently been FDA-approved for clinical use in evaluating mTBI (43). However, challenges in clinical translation persist, highlighting the need for novel biomarkers. Epigenetic mechanisms, including small non-coding RNAs (sncRNAs), DNA methylation, and histone modifications, have emerged as critical regulators of the post-injury response. miRNAs and snoRNAs are increasingly recognized as modulators of neuroinflammation and neuroprotection, while reciprocal regulation between miRNAs and epigenetic modifiers (e.g., DNA methyltransferases, histone deacetylases) suggests a dynamic network shaping long-term injury outcomes (44).

Within this context, several miRNAs, such as miR-21, miR-92a, miR-155, and members of the let-7 family, have shown altered expression following TBI and have been linked to processes including inflammation, metabolism, and neuronal survival (45). Importantly, miRNAs are highly stable in biological fluids and resistant to enzymatic degradation and freeze–thaw cycles, making them attractive candidates for non-invasive diagnostics (45). While emerging evidence supports their role as biomarkers, the contribution of let-7 specifically to TBI pathophysiology remains poorly defined. To clarify this relationship, the present review systematically evaluates the evidence for the differential expression of let-7 family members in TBI. It explores their potential role as both biomarkers and mechanistic mediators of injury outcomes.

To clarify current understanding, this systematic review examined the literature to answer the question: *Which let-7 family members are differentially expressed in TBI?*

## 2 Materials and methods

This systematic review was registered with PROSPERO in 2025, CRD420251129282. Available from https://www.crd.york.ac.uk/PROSPERO/view/CRD420251129282.

### 2.1 Systematic review

A systematic review was undertaken using the Preferred Reporting Items for Systematic Reviews and Meta-Analyses (PRISMA) guidelines and the PRISMA checklist (46, 47). The research question, “To identify the differentially expressed let-7 family members in TBI,” enabled the generation of focused keywords that were used to search and retrieve relevant records from three databases. Explicit inclusion and exclusion criteria were developed and used to complete an abstract. The full-text screening of the records was conducted to determine those eligible for inclusion. This process was done by hand by a primary reviewer, who included articles that were subsequently checked by a second reviewer (NR, VDP).

### 2.2 Search strategy

The Population, Intervention, Comparison(s), and Outcome(s) (PICOs) framework for systematic reviews was utilized to define and focus the research question. This Cochrane Collaboration-recommended system ensures that the full scope of evidence is considered within defined parameters, and a quantitative investigation of the results can be undertaken.

1) Population: All animal species.
2) Intervention: TBI. Human participants will be assessed by a clinician, and any validated TBI animal model will be included.
3) Comparisons: Healthy age-matched controls with no TBI or sham animals.
4) Outcomes: The fold change in miRNA expression profiles between TBI and controls.

### 2.3 Search terms and databases

The following keywords were selected to represent the PICO criteria and combined with Boolean operators to generate the string search: (Traumatic Brain Injury OR TBI OR concussion) AND (let-7a OR let-7b OR let-7c OR let-7d OR let-7e OR let-7f OR let-7g OR let-7i OR mir-98 OR miR-202). No filters were applied. The search was carried out in three databases: PubMed, EMBASE through Ovid, and Web of Science. The records retrieved were collated in EndNote 20 (Clarivate, Philadelphia, PA, USA), where they were screened for duplicates, and any identified were removed. The remaining abstracts were then manually assessed for eligibility by the two independent reviewers using our predefined inclusion and exclusion criteria outlined in Table 2.

**Table 2:** Criteria of exclusion and inclusion of papers in this systematic review.

**Inclusion criteria**	**Exclusion criteria**
Published in English	Studies published not in English
Primary research published in peer-reviewed journals	Non-primary data articles, Conference abstracts, commentaries, editorials, and letters
Traumatic Brain Injury, all severities and time-points	Other neuropathology
Human and animal studies	*In vitro* model, cultured cells
Brain tissue and all biofluids	None
Qualitative and quantitative microRNA expression analysis, including at least one member of the let-7 family	Studies focusing on post-translational modifications, mutations/allelic variants, and intervention

### 2.4 Data extraction

Data were extracted from the final included studies and imported into Microsoft Excel (Microsoft Corporation, Redmond, WA, USA) (Table 1). The title of the article, authors, and abstract summaries were saved to identify the studies. The species, tissue, TBI severity, TBI model, time points, and results, including let-7 up- or down-regulation compared to control groups, were recorded.

### 2.5 Risk of bias

Risk of bias was assessed using two different tools, the Evidence Project risk of bias tool for human studies (48) and the SYRCLE risk of bias (RoB) tool for animal studies (49). Risk for each study was assessed across the different domains by two independent reviewers (VDP and GL), with any disagreements being settled through discussion. The Evidence Project risk of bias tool is a tool for assessing the risk of bias across both randomized and non-randomized study designs. The tool consists of eight items, each assessed as either present (yes) or absent (no), some of which are also marked as not applicable or not reported. The items are as follows: (1) cohort, (2) control or comparison group, (3) pre-post intervention data, (4) random assignment of participants to the intervention, (5) random selection of participants for assessment, (6) follow-up rate of 80% or higher, (7) equivalence of comparison groups in socio-demographics, and (8) equivalence of comparison groups at baseline regarding outcome measures. Items (1)–(3) provide an overview of the study design, while the other items address additional aspects of study rigor. The SYRCLE risk of bias (RoB) tool for animal studies instead used the following domains to assess the potential risk of bias across the studies: random sequence generation, baseline characteristics described, correct timing of randomization, allocation concealment, random housing, blinding, random outcome assessment, incomplete data, sample size calculation, and primary outcome specified.

## 3 Results

### 3.1 Systematic review

**Figure 2:**
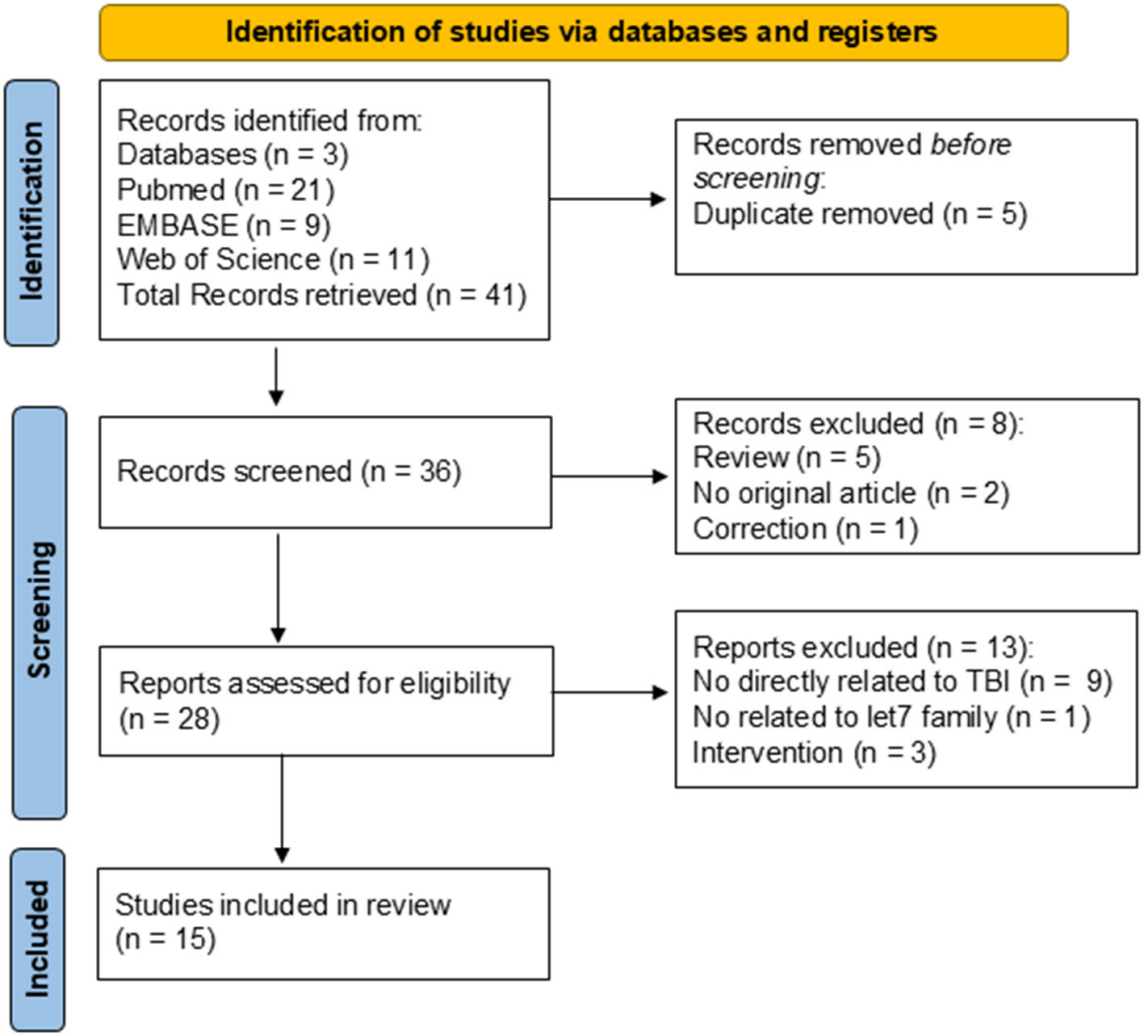
PRISMA diagram of systematic review search.

Electronic searches of PubMed, EMBASE, and Web of Science generated 21, 9, and 11 records, respectively, making a total of 41 records. After duplicate removal, 36 abstracts were screened, and *n* = 8 were excluded because of the review or the abstract. Twenty-eight full-text records were assessed for eligibility, and 15 papers met all the criteria and were included in this review. Causes for exclusion were: no direct relationship to TBI (*n* = 9), no relationship to the let-7 family (*n* = 1), and intervention (*n* = 3). The PRISMA diagram is presented in Figure 2.

### 3.2 Data extraction

The primary data were extracted from each study and listed in Table 3, showing references, species, tissues, TBI severities or models, time points for assessment, and expression of let-7 family members.

**Table 3:** Data extraction.

**Authors**	**Species**	**Tissue**	**Type of injury**	**Time points**	**Results**
Wyczechowska et al. (50)	Human	Serum	Concussion	2 h;18 h; end of season	let-7c (↑ 2 h+18 h vs. end of season); let-7c (↑ 2 h vs. end of season; ↑ 18 h vs. end of season); let-7b (↑ 18 h vs. 2 h and end of season)
Svingos et al. (51)	Human	Serum	Concussion	Baseline; 4 h	let-7a (↑ 4 h)
Ma et al. (52)	Human	Serum	Severe TBI	2 h; 12 h; 24 h; 48 h; 72 h	let-7b and let-7f (↑ 12hvs2h); let-7a (↓12 h vs. 2 h); let-7f (↑ 24 h vs. 2 h); let-7a (↓24 h vs. 2 h); let-7b (↑ 48 h vs. 2 h); let-7f (↑ 72 h vs. 2 h)
Mitra et al. (53)	Human	Plasma	Mild TBI	28 days from injury	No changes in let-7f
Petrova et al. (54)	Human	CSF, plasma	Severe TBI	from 2 M up to 2 Y with or without TBI	Let-7b (↓CSF); No changes in plasma
Seršić et al. (55)	Human	CSF	Severe TBI	D 1-2, D 3-4, D 5-6, and D 7-12 CSF pools	let-7a, b, c, d, e, f, g, i (↓ over the time-points)
Di Pietro et al. (56)	Human	Saliva	Concussion	48 h/72 h	let-7i (↑ 36 h)
Johnson et al. (58)	Human	Saliva	Concussion	14 D; 4 W	let-7f (↑ PCS vs. ACS); let-7a associated with prolonged symptoms; let-7b associated with fatigue
Di Pietro et al. (57)	Human	Saliva	Concussion	In-match; post-match; 36 h-48 h	let-7a, b, i, f (↑ in concussion vs. different controls)
Lv et al. (59)	Mouse	Brain	CCI (impact velocity at 3.5 m/s, deformation depth at 2.0 mm, and duration for 150 ms)	from 6 h to 5 D	let-7c (↓ from 6 h to 5 days)
Miao et al. (60)	Mouse	Brain	CCI (impact velocity 4.5 m/s, deformation depth at 1.5 mm)	3 W	let-7c (↓)
He et al. (61)	Mouse	Brain	HSI (2.5 mm posterior to bregma, 2.3 mm lateral to the sagittal suture, and 3 mm below the dura)	3 D, 7 D, 14 D	let-7c (↓3D); let-7i (↓3D, 7D);
Johnson et al. (62)	Rat	Brain, serum	Blast Injury (unilateral frontal 10% PBB)	4 h; 7 D	let-7i (↑ 4 h, brain); let-7i (↓7 D, serum);
Balakathiresan et al. (63)	Rat	Serum, CSF	Blast Injury (2.5-ft compression chamber connected to a 15-ft expansion chamber). SSI: three serial BOP of 120 kPa at an interval of 2 h; LII: 3 BOP of 120 kPa at an interval of 24 h	3 h and 24 h after the last BOP exposure	let-7i (↑ in Serum and CSF of SSI at both time-points)
Sajja et al. (64)	Mouse	Plasma	Blast injury (mild to moderate; 17, 17 × 3, and 20 psi blast)		let-7 a, b, g (↓ 17 x 3 psi)

CCI, Controlled Cortical Impact; HIS, hippocampal stab injury; h, hours; D, day; W, week; M, month; Y, year.

### 3.3 Risk of bias

The Evidence Project risk of bias tool indicated that five are cohort studies, and most of the studies have a control or comparison group. Only three studies collected data pre- and post-intervention. Random assignment of participants to the intervention was not applicable for all studies; while datasets were nearly complete, the equivalent comparison groups on socio-demographics or baseline disclosure varied across studies. This variation resulted in a high overall risk of bias for four studies, an unclear risk for two, and a low risk for the remaining three (Table 4). RoB analysis found that all included articles, referring to data obtained in experimental TBI in laboratory animals, specified the primary outcomes and described baseline characteristics. All studies had full data availability with no missing data. However, for many of them, it is unclear whether the randomly housed animals or concealed treatment allocations. Incidents of random sequence generation, blinding, and random outcome assessment were not always specified. Thus, the overall risk of bias was judged to be unclear for three studies, potentially invalidating certain findings of the included papers, and low for the remaining three studies. RoB analysis results are depicted in the table below (Table 5).

**Table 4:** Risk of bias assessment according to the Evidence Project risk of bias tool.

**Criteria**	**Wyczechowska et al. (50)**	**Svingos et al. (51)**	**Ma et al. (52)**	**Mitra et al. (53)**	**Petrova et al. (54)**	**Seršić et al. (55)**	**Di Pietro et al. (56)**	**Johnson et al. (58)**	**Di Pietro et al. (57)**
Cohort	No	Yes	Yes	Yes	No	Yes	No	No	Yes
Control or comparison group	Yes	Yes	Yes	Yes	Yes	No	Yes	Yes	Yes
Pre/Post intervention data	No	Yes	No	Yes	No	No	No	No	Yes
Random assignment of participants to the intervention	NA	NA	NA	NA	NA	NA	NA	NA	NA
Random selection of participants for assessment	Yes	Yes	Yes	Yes	Yes	Yes	Yes	Yes	Yes
Follow-up rate of 80% or more	No	Yes	Yes	Yes	Yes	No	NA	Yes	Yes
Comparison groups equivalent in sociodemographic	Yes	Yes	No	NR	NR	NA	No	Yes	Yes
Comparison groups equivalent at baseline on disclosure	No	Yes	No	Yes	No	No	Yes	No	Yes
Overall Risk of Bias	High	Low	Unclear	Low	High	High	High	Unclear	Low

NR, not reported; NA, not applicable.

**Table 5:** Risk of bias assessment according to the SYRCLE risk of bias (RoB) tool for animal studies.

**Criteria**	**Lv et al. (59)**	**Miao et al. (60)**	**He et al. (61)**	**Johnson et al. (62)**	**Balakathiresan et al. (63)**	**Sajja et al. (64)**
Was the allocation sequence adequately generated and applied?	Yes	Unclear	Unclear	Unclear	Unclear	Unclear
Were the groups similar at baseline, or were they adjusted for confounders in the analysis?	Yes	Yes	Yes	Yes	Yes	Yes
Was the allocation to the different groups adequately concealed during?	Unclear	Unclear	Unclear	Unclear	Unclear	Unclear
Were the animals randomly housed during the experiment?	Unclear	Unclear	Yes	Yes	Unclear	Unclear
Were the caregivers and/or investigators blinded from knowledge of which intervention each animal received during the experiment?	Yes	No	Yes	Unclear	Unclear	Unclear
Were animals selected at random for outcome assessment?	Unclear	Unclear	Unclear	Unclear	Unclear	Unclear
Was the outcome assessor blinded?	Yes	Yes	Yes	Unclear	Unclear	Unclear
Were incomplete outcome data adequately addressed?	Yes	Yes	Yes	Yes	Yes	Yes
Are reports of the study free of selective outcome reporting?	Yes	Yes	Yes	Yes	Unclear	Yes
Was the study apparently free of other problems that could result in a high risk of bias?	Yes	Yes	Yes	Unclear	Unclear	Yes
Overall Risk of Bias	Low	Low	Low	Unclear	Unclear	Unclear

### 3.4 Human studies

Wyczechowska and Svingos have investigated the expression of specific microRNAs in the blood of small groups of collegiate football players. Wyczechowska et al. (50) examined 21 athletes (18–23 years of age) over an entire practice and game season. Of them, 10 experienced a concussion, and the remaining 11 non-concussed athletes were used as the matched control group. Authors found that serum let-7c-5p increased in the concussed group during the acute phase post-injury (at 2 and 18 h) and demonstrated that circulating let-7c-5p levels had high specificity and sensitivity for distinguishing concussed from non-concussed players. Additionally, it was found that serum let-7b-3p was also elevated at 18 h post-injury. In the second study, Svingos et al. (51) studied 27 collegiate athletes, with a mean age of 18.8 ± 0.8 years, who were analyzed for let-7 levels in serum from peripheral blood before the beginning of the season (baseline) and within 4 h post-concussion. The levels of let-7a-5p showed a significant increase when comparing post-concussion measurements to the baseline values. In another study, Ma et al. (52) used serum as the sample of choice for let-7 determination. They selected circulating microRNA expression, which was evaluated in 20 patients (aged 32–56 years) at different times (2, 12, 24, 48, and 72 h) following severe TBI. The authors found that whilst let-7a was downregulated, let-7b and let-7f were upregulated. Finally, Mitra et al. found no changes in let-7f between individuals with mTBI who report post-concussive symptoms at the 28-day mark and those who do not (53).

Petrova and Seršić focused on cerebrospinal fluid (CSF) for the evaluation of let-7 levels following head injury. Petrova et al. (54) measured hsa-let-7b-5p in both CSF and blood of patients affected by prolonged disorders of consciousness (lasting from 2 months to 2 years) caused by prior severe brain damage. In 22 of them, the previous brain injury was caused by a severe TBI. Compared to the control group (10 patients undergoing surgical operations due to extracerebral volumetric brain neoplasms, meningiomas, and basilar artery aneurysms), it was found that the subgroup of severely injured TBI patients had an upregulation of let-7b in CSF but not in blood. Seršić et al. (55) analyzed 35 CSF samples from five patients with severe TBI over 12 days post-injury, pooling samples into intervals (days 1–2, 3–4, 5–6, and 7–12). They targeted 87 miRNAs and found high levels for many of them, including let-7a, b, c, d, e, f, g, and i, on the first day of post-injury, with a progressive decline for longer times after severe TBI.

Finally, three studies on concussed patients used saliva as the body fluid in which to perform the quantification of let-7. Di Pietro et al. conducted two of these studies. In 2018 (56), they collected saliva samples from 22 concussed athletes and 10 matched controls 48–72 h post-concussion, finding that let-7i-5p was significantly upregulated in the concussed group. A larger follow-up study in 2021 (57) involved male professional players in England's top rugby union tiers across two seasons (2017–2019). Samples were taken pre-season from 1,028 players and during standardized head injury assessments (HIAs) from 156 players at three time points (in-game, post-game, and 36–48 h post-game), along with samples from 102 uninjured players and 66 with musculoskeletal injuries. This study revealed increases in let-7a, b, i, and f in the saliva of concussed players compared to the different control groups, with let-7f-5p showing the highest area under the curve (AUC) at 36–48 h.

In the third study, Johnson et al. (58) evaluated the efficacy of measuring salivary microRNAs in identifying children at risk for prolonged concussion symptoms. In this cohort study of 52 patients aged 7–21 years, microRNAs were measured within 14 days after head injury and at 4 and 8 weeks following concussion. It was found that there was upregulation of salivary let-7f in patients with post-concussion syndrome (PCS) compared to those with acute concussion. Additionally, let-7a-3p accurately identified patients with prolonged symptoms, while high salivary levels of let-7b were associated with fatigue.

### 3.5 Animal studies

Three studies have indicated a decrease in let-7-5p levels in TBI-injured mouse brains. Lv et al. (59) demonstrated that let-7c-5p levels dropped rapidly after controlled cortical impact (CCI) TBI and gradually returned to baseline by 14 days post-injury. Miao et al. observed a downregulation of let-7c following TBI induced by a pneumatic device, with variations noted in mice that engaged in voluntary exercise prior to the injury (60). Additionally, He et al. (61) reported a reduction in let-7c, 3 days after a hippocampal stab injury (HSI), also noting downregulation of let-7i at 2- and 7-days post-injury. Johnson et al. (62) examined let-7i in a rat model of penetrating ballistic-like brain injury (PBBI), collecting ipsilateral brain tissue and serum from 4 h to 7 days post-injury. They found that brain tissue levels of let-7i increased at 4 h, while serum let-7i decreased in serum at 7 days after PBBI. Balakathiresan et al. (63) investigated serum and CSF levels of let-7i following post-blast wave exposure in two injury groups: a short interval injury group (SII), which was exposed to three serial blasts at 120 kPa every 2 h, and a long interval injury group (LII), where blasts were administered every 24 hours. Samples from both groups were collected at 3 and 24 h after the last blast, revealing the presence of let-7i in both serum and CSF immediately after injury. Sajja et al. (64) utilized a murine model of mild-to-moderate blast-induced TBI, exposing animals to blast intensities of 17, 17 × 3, and 20 psi using a calibrated blast simulator. The study found that plasma levels of brain-enriched miRNAs let-7a, b, and g were reduced at 17 × 3 and 20 psi, and let-7c was downregulated at 17 × 3 only, while let-7d levels increased in the 17 psi group.

## 4 Discussion

This systematic review revealed the involvement of the let-7 family in the pathobiological mechanisms of TBI across varying severities and time points. Data from experimental TBI in laboratory animals and humans have notably highlighted that the different expression patterns depend on whether let-7 levels are measured in brain tissue (animal studies) or biofluids (animal and clinical studies).

Human studies showed an overall upregulation of let-7 family members across different biofluids, such as plasma, serum, and saliva, regardless of trauma severity (ranging from concussions to severe TBIs), population demographics (athletes vs. the general population), age, gender, or time since injury (from one day post-injury onward). The only exception was in CSF, where downregulation was predominant. In contrast, animal studies primarily demonstrated a downregulation of let-7 expression in brain tissue, with only a few exceptions. This pattern is not unique to TBI and aligns with findings in other diseases, such as cancer, where tissue levels of let-7 are generally low, but levels in the bloodstream are elevated, suggesting its potential as a biomarker (65–67). The inverse trend between tissue and biofluid levels may indicate a common regulatory mechanism for microRNAs, independent of the pathology considered, where cells release microRNAs to decrease their intracellular concentration, resulting in elevated levels in biofluids.

Based on the analysis of available literature, it can be stated that human studies have primarily focused on the use of let-7 as a biomarker for diagnostic and prognostic purposes. However, neither goal has been achieved to date, since (i) no correlation between let-7 levels in biofluids and TBI severity has been found, and (ii) no clear data have yet been produced correlating let-7 levels in biofluids with TBI patients' outcome.

Results of animal studies have explored more mechanistic aspects of the potential roles of let-7 variations in TBI. In the intricate landscape of one of the most complex and challenging pathologies affecting the brain, the let-7 family emerges as a crucial player in orchestrating the protective responses of nervous cells following TBI. Let-7 appears to play a crucial role in modulating the inflammatory cascade that typically follows TBI, which can both harm and help CNS recovery. Neuroinflammation is mainly driven by microglia and infiltrating macrophages, which amplify inflammation by releasing soluble cellular components in the injured brain (68, 69). Microglia, acting as the first responders to CNS injuries, can adopt either a “classically activated” M1 phenotype or an “alternatively activated” M2 phenotype (70, 71). While M1 polarization is associated with neuronal dysfunction and cell death, M2 polarization promotes anti-inflammatory responses, aids in debris clearance, and supports CNS repair. Modifying the M2/M1 ratio has shown promising therapeutic effects in TBI (72). MicroRNAs have been implicated in this modulation, with let-7c being one of the most abundant and conserved miRNAs linked to macrophage polarization (73, 74). Lv et al. (59) showed that let-7c was rapidly reduced in traumatically injured foci in the brains of adult C57BL/6J mice. The intracerebroventricular injection (ICV) of let-7c improved the outcomes of mice subjected to CCI by inhibiting neuroinflammation. In particular, the attenuation of microglia/macrophage activation was obtained through the inhibition of M1 polarization and enhancement of M2 polarization. In addition, authors showed that expressions of the let-7c putative target gene caspase-3 and of PKC-d (a modulator of caspase-3 effects) were inhibited. Miao et al. (60) also found that let-7c could be involved in the prevention and protection afforded by voluntary exercise in a mouse model of TBI induced by a pneumatic impact device. They showed that voluntary running wheel (RW) exercise prior to TBI significantly reduced the mortality rate and righting time in injured mice. They also showed that, following TBI, brain levels of let-7c were significantly different in the group of exercised mice compared to those of the untrained. Therefore, the authors proposed that an epigenetic mechanism, capable of inducing amelioration of functional deficits, might be involved in the beneficial effects of voluntary RW exercise following TBI.

It appears that let-7 can also help in minimizing TBI-associated secondary injury, particularly the neuroinflammatory processes caused by the release of pro-inflammatory cytokines (like TNF-α and IL-6), which can inadvertently cause further tissue damage. In this light, Johnson et al. used the rats' penetrating ballistic-like brain injury (PBBI) model to determine if changes in microRNA levels, including let-7i, were connected to those of neuroinflammatory mediators. Results showed an inverse correlation between let-7i and IL6, an interaction that was also previously described (75), suggesting let-7i as a plausible therapeutic target of TBI-induced inflammation. Hence, let-7 serves to mitigate the TBI-exacerbated neuroinflammatory response and directs immune cells toward protective functions. In this context, He et al. not only showed let-7i downregulation in a model of HSI in mice but also found that non-invasive intranasal delivery of let-7i significantly improves cognitive function and suppresses neuroinflammation, glial scar formation, and neuronal apoptosis via inhibition of the stimulator of interferon genes (STING). STING is a key regulator of inflammation, and its expression was found to be elevated in postmortem human TBI brains (76, 77). Balakathiresan et al. (63) and Sajja et al. (64) instead limited their studies to investigating microRNAs as potential biomarkers of TBI. They both found several members of the let-7 family differentially expressed in the CSF and serum of rodents undergoing blast injury.

However, since let-7′s roles are not limited to modulating mechanisms of inflammation, it is evident that the information obtained so far, in both experimental and clinical studies in TBI, is certainly not sufficient. For instance, several indications have been produced showing that the activity of the PDH complex, the key multi-enzymatic system ensuring the oxidation of pyruvate to form acetyl-CoA and thus fueling the Krebs cycle, is deeply modulated by let-7 through its action on the gene expressing the main PDH complex inhibitor (PDK, pyruvate dehydrogenase kinase). Using Marmarou's model of closed-head weight drop TBI in the rat, it was shown that profound glucose dysmetabolism (32) is associated with the PDH complex inhibition because of overexpression of PDK and concomitant downregulation of PDP (pyruvate dehydrogenase phosphorylase, the main activator of PDH) (78).

Additionally, although obtained by bioinformatic data only, it has also been indicated that let-7 may influence the homeostasis of N-acetylaspartate (NAA), the main neuron-specific N-acetylated amino acid deeply altered by TBI (79). Since the metabolism of NAA is strictly dependent on acetyl-CoA bioavailability (this compound is the acetyl group donor in the NAA synthesis catalyzed by NAT8L), it is easy to hypothesize that the let-7 modulation of both ASPA and PDK may have profound consequences on brain metabolism following TBI.

It should not, however, be disregarded that let-7 family members can influence neurogenesis. Particularly, the differentiation of neural stem cells into neurons in the TBI-injured brain may be highly relevant in aiding the replacement of lost or damaged cells after TBI. It should even be worth investigating whether they play a role in enhancing synaptic plasticity, which is crucial for functional recovery in cognitive tasks of the post-injured brain. Among the other potential roles of let-7 in the post-injury brain, the mitigation of oxidative/nitrosative stress, one of the mechanisms of nervous cell damage potently activated by TBI, might certainly be better characterized. Indications obtained under different pathological conditions, but TBI, suggest that let-7 is mutually regulated by FOXO3 and PRDX1 expression levels, both involved in the cellular capacity to maintain a correct oxido-reductive state of -SH groups of cysteine, deeply altered under conditions of oxidative/nitrosative stress (80). Also in this case, it has been shown that TBI deeply alters the cerebral concentrations of GSH, the main cellular antioxidant specific for keeping free -SH groups in the correct oxido-reductive state (32, 37), thereby rendering plausible the involvement of let-7 in the overall damaging process mediated by excess production of ROS and RNS occurring following TBI. Modulation of PI3K/Akt and MAPK pathways, thus promoting cell survival and growth, reinforces the brain's resilience in the wake of injury.

In the aftermath of TBI, the let-7 family serves as a multifaceted protector, navigating the complex interplay of inflammation, mitochondrial-related energy production, oxidative/nitrosative stress, and regeneration; each of them, if positively affected by let-7 modulation, may greatly improve nervous cell survival and, potentially, the outcome of TBI patients. Their contributions are vital for healing, illuminating pathways for future therapies that could help countless individuals reclaim their lives after the devastation of brain injury.

## 5 Limitations

The present research highlighted the promise of let-7 as both a biomarker and a therapeutic target. Changes in its expression can indicate the severity of TBI, offering insights into patient prognosis. Moreover, the potential to manipulate let-7 levels, through mimics or inhibitors, opens new avenues for treatment, aiming to harness its protective capabilities and enhance recovery. However, the use of miRNA signatures as a novel diagnostic and prognostic tool remains largely in the descriptive phase yet. While a wealth of data has been gathered across various disease states, translating this information into clinical applications requires larger studies and universally accepted guidelines. One major issue is the need for standardized protocols for miRNA extraction, quantification, and analysis. Variability in sample collection, processing methods, and analytical techniques can lead to inconsistent results, hindering the establishment of universally accepted miRNA biomarkers. A shared information infrastructure for data exchange, analysis, and protocol standardization would greatly enhance research in miRNA biomarker discovery. Furthermore, the biological complexity of miRNA regulation necessitates a deeper understanding of their roles in specific contexts. For instance, miRNA expression can be influenced by factors such as age, gender, lifestyle, and environmental conditions. Therefore, identifying well-characterized endogenous miRNAs that are consistent across different populations and conditions is crucial for their clinical applicability.

Regarding animal models of TBI, these are essential for understanding the underlying mechanisms and potential treatments, yet the variety of methodologies and tissues analyzed can lead to discrepancies in results. Methodologically, injuries can be induced through various techniques, including controlled cortical impact, fluid percussion, weight drop, or blast overpressure, leading to differences in injury severity and patterns. Additionally, the specific tissues analyzed, ranging from the brain cortex to subcortical structures and the cerebrospinal fluid, can yield varying insights into the pathophysiological changes following TBI. For example, while some studies focus on acute inflammatory responses in the cortex, others might assess long-term neurodegenerative changes in deeper brain regions. This variability can complicate comparisons across studies and hinder the establishment of a consensus on the biological mechanisms involved in TBI, highlighting the need for standardized protocols and comprehensive analyses.

Importantly, our review is limited by the quality of available evidence. Of the 15 studies included, 9 showed an unclear or high risk of bias. This limitation significantly affects the strength of our conclusions and underscores the need for further high-quality, standardized, and rigorous research in this field. Establishing universally accepted guidelines and infrastructure for data sharing and analysis will be crucial to advancing miRNA-based diagnostics and therapeutics in TBI.

## 6 Conclusions

We found that the let-7 family of microRNAs plays a multifaceted role in the response to TBI. By regulating inflammation, promoting neuronal survival, enhancing neurogenesis, and modulating key signaling pathways, let-7 microRNAs play a crucial role in mitigating damage and facilitating recovery. Their potential as biomarkers and therapeutic targets highlights their importance in advancing TBI treatment strategies. miRNAs may soon become invaluable tools in clinical practice, revolutionizing the way we approach disease management and patient care. Further research into their specific mechanisms and effects may lead to innovative approaches for improving outcomes in TBI patients; however, such research is mandatory.

## References

[1] Reinhart BJ Slack FJ Basson M Pasquinelli AE Bettinger JC Rougvie AE. The 21-nucleotide let-7 RNA regulates developmental timing in Caenorhabditis elegans. Nature. (2000) 403:901–6. 10.1038/35002607 10706289

[2] Pasquinelli AE Reinhart BJ Slack F Martindale MQ Kuroda MI Maller B. Conservation of the sequence and temporal expression of let-7 heterochronic regulatory RNA. Nature. (2000) 408:86–9. 10.1038/35040556 11081512

[3] Ali A Bouma GJ Anthony RV Winger QA. The Role of LIN28. Int J Mol Sci. (2020) 21:3637. 10.3390/ijms21103637 32455665 PMC7279312

[4] Hertel J Bartschat S Wintsche A Otto C Stadler PF. Lab SotBC. Evolution of the let-7 microRNA family. RNA Biol. (2012) 9:231–41. 10.4161/rna.18974 22617875 PMC3384580

[5] Bernstein DL Jiang X Rom S. microRNAs: their role in cerebral and cardiovascular diseases, inflammation, cancer, and their regulation. Biomedicines. (2021) 9:606. 10.3390/biomedicines9060606 34073513 PMC8227213

[6] Ruby JG Jan C Player C Axtell MJ Lee W Nusbaum C. Large-scale sequencing reveals 21U-RNAs and additional microRNAs and endogenous siRNAs in C. elegans Cell. (2006) 127:1193–207. 10.1016/j.cell.2006.10.040 17174894

[7] Büssing I Slack FJ Grosshans H. let-7 microRNAs in development, stem cells and cancer. Trends Mol Med. (2008) 14:400–9. 10.1016/j.molmed.2008.07.001 18674967

[8] Lee H Han S Kwon CS Lee D. Biogenesis and regulation of the let-7 miRNAs and their functional implications. Protein Cell. (2016) 7:100–13. 10.1007/s13238-015-0212-y 26399619 PMC4742387

[9] Komarovsky Gulman N Armon L Shalit T Urbach A. Heterochronic regulation of lung development. FASEB J. (2019) 33:12008–18. 10.1096/fj.201802702R 31373834 PMC6902705

[10] Fairchild CLA Cheema SK Wong J Hino K Simó S La Torre A. Let-7 regulates cell cycle dynamics in the developing cerebral cortex and retina. Sci Rep. (2019) 9:15336. 10.1038/s41598-019-51703-x 31653921 PMC6814839

[11] Morgado AL Rodrigues CM Solá S. MicroRNA-145 regulates neural stem cell differentiation through the Sox2-Lin28/let-7 signaling pathway. Stem Cells. (2016) 34:1386–95. 10.1002/stem.2309 26849971

[12] Patterson M Gaeta X Loo K Edwards M Smale S Cinkornpumin J. let-7 miRNAs can act through notch to regulate human gliogenesis. Stem Cell Reports. (2014) 3:758–73. 10.1016/j.stemcr.2014.08.015 25316189 PMC4235151

[13] Xiang W Tian C Peng S Zhou L Pan S Deng Z. Let-7i attenuates human brain microvascular endothelial cell damage in oxygen glucose deprivation model by decreasing toll-like receptor 4 expression. Biochem Biophys Res Commun. (2017) 493:788–93. 10.1016/j.bbrc.2017.08.093 28844675

[14] Isanejad A Alizadeh AM Amani Shalamzari S Khodayari H Khodayari S Khori V. MicroRNA-206, let-7a and microRNA-21 pathways involved in the anti-angiogenesis effects of the interval exercise training and hormone therapy in breast cancer. Life Sci. (2016) 151:30–40. 10.1016/j.lfs.2016.02.090 26924493

[15] Wang T Wang G Hao D Liu X Wang D Ning N. Aberrant regulation of the LIN28A/LIN28B and let-7 loop in human malignant tumors and its effects on the hallmarks of cancer. Mol Cancer. (2015) 14:125. 10.1186/s12943-015-0402-5 26123544 PMC4512107

[16] Chen Y Xie C Zheng X Nie X Wang Z Liu H. LIN28/. Cancer Immunol Res. (2019) 7:487–97. 10.1158/2326-6066.CIR-18-0331 30651289

[17] Li XX Di X Cong S Wang Y Wang K. The role of let-7 and HMGA2 in the occurrence and development of lung cancer: a systematic review and meta-analysis. Eur Rev Med Pharmacol Sci. (2018) 22:8353–66. 30556876 10.26355/eurrev_201812_16533

[18] Tsang WP Kwok TT. Let-7a microRNA suppresses therapeutics-induced cancer cell death by targeting caspase-3. Apoptosis. (2008) 13:1215–22. 10.1007/s10495-008-0256-z 18758960

[19] Zha W Guan S Liu N Li Y Tian Y Chen Y. Let-7a inhibits Bcl-xl and YAP1 expression to induce apoptosis of trophoblast cells in early-onset severe preeclampsia. Sci Total Environ. (2020) 745:139919. 10.1016/j.scitotenv.2020.139919 32721616

[20] Zhang H Xiong X Gu L Xie W Zhao H. CD4 T cell deficiency attenuates ischemic stroke, inhibits oxidative stress, and enhances Akt/mTOR survival signaling pathways in mice. Chin Neurosurg J. 2018; 4. 10.1186/s41016-018-0140-9 32832192 PMC7398241

[21] Bao B Ali S Ahmad A Li Y Banerjee S Kong D. Differentially expressed miRNAs in cancer-stem-like cells: markers for tumor cell aggressiveness of pancreatic cancer. Stem Cells Dev. (2014) 23:1947–58. 10.1089/scd.2013.0551 24734907

[22] Iliopoulos D Hirsch HA Struhl K. An epigenetic switch involving NF-kappaB, Lin28, Let-7 MicroRNA, and IL6 links inflammation to cell transformation. Cell. (2009) 139:693–706. 10.1016/j.cell.2009.10.014 19878981 PMC2783826

[23] Zhu H Shyh-Chang N Segrè AV Shinoda G Shah SP Einhorn WS. The Lin28/let-7 axis regulates glucose metabolism. Cell. (2011) 147:81–94. 10.1016/j.cell.2011.08.033 21962509 PMC3353524

[24] Ma X Li C Sun L Huang D Li T He X. Lin28/let-7 axis regulates aerobic glycolysis and cancer progression via PDK1. Nat Commun. (2014) 5:5212. 10.1038/ncomms6212 25301052

[25] Peng F Wang JH Fan WJ Meng YT Li MM Li TT. Glycolysis gatekeeper PDK1 reprograms breast cancer stem cells under hypoxia. Oncogene. (2018) 37:1119. 10.1038/onc.2017.407 29251717 PMC5851083

[26] Zhang Y Li C Hu C Wu Q Cai Y Xing S. Lin28 enhances *de novo* fatty acid synthesis to promote cancer progression via SREBP-1. EMBO Rep. (2019) 20:e48115. 10.15252/embr.201948115 31379107 PMC6776893

[27] Huang S Lv Z Guo Y Li L Zhang Y Zhou L. Identification of Blood Let-7e-5p as a Biomarker for Ischemic Stroke. PLoS One. (2016) 11:e0163951. 10.1371/journal.pone.0163951 27776139 PMC5077157

[28] Langlois JA. The epidemiology and impact of traumatic brain injury: a brief overview. J Head Trauma Rehabil. (2006) 21:375–8 10.1097/00001199-200609000-00001 16983222

[29] Rubiano AM Carney N Chesnut R Puyana JC. Global neurotrauma research challenges and opportunities. Nature. (2015) 527:S193–7. 10.1038/nature16035 26580327

[30] Brown AW Moessner AM Mandrekar J Diehl NN Leibson CL Malec JF. survey of very-long-term outcomes after traumatic brain injury among members of a population-based incident cohort. J Neurotrauma. (2011) 28:167–76. 10.1089/neu.2010.1400 21121813 PMC3064530

[31] Di Pietro V Amorini AM Tavazzi B Hovda DA Signoretti S Giza CC. Potentially neuroprotective gene modulation in an *in vitro* model of mild traumatic brain injury. Mol Cell Biochem. (2013) 375:185–98. 10.1007/s11010-012-1541-2 23242602

[32] Di Pietro V Lazzarino G Amorini AM Tavazzi B D'Urso S Longo S. Neuroglobin expression and oxidant/antioxidant balance after graded traumatic brain injury in the rat. Free Radic Biol Med. (2014) 69:258–64. 10.1016/j.freeradbiomed.2014.01.032 24491879

[33] Amorini AM Lazzarino G Di Pietro V Signoretti S Belli A Tavazzi B. Metabolic, enzymatic and gene involvement in cerebral glucose dysmetabolism after traumatic brain injury. Biochim Biophys Acta. (2016) 1862:679–87. 10.1016/j.bbadis.2016.01.023 26844378

[34] Di Pietro V Lazzarino G Amorini AM Signoretti S Hill LJ Porto E. Fusion or fission: the destiny of mitochondria in traumatic brain injury of different severities. Sci Rep. (2017) 7:9189. 10.1038/s41598-017-09587-2 28835707 PMC5569027

[35] Lazzarino G Mangione R Saab MW Tavazzi B Pittalà A Signoretti S. Traumatic brain injury alters cerebral concentrations and redox states of coenzymes Q. Antioxidants. (2023) 12:985. 10.3390/antiox12050985 37237851 PMC10215158

[36] Hovda DA Lee SM Smith ML Von Stuck S Bergsneider M Kelly D. The neurochemical and metabolic cascade following brain injury: moving from animal models to man. J Neurotrauma. (1995) 12:903–6. 10.1089/neu.1995.12.903 8594218

[37] Di Pietro V Yakoub KM Caruso G Lazzarino G Signoretti S Barbey AK. Antioxidant therapies in traumatic brain injury. Antioxidants. (2020) 9:260. 10.3390/antiox9030260 32235799 PMC7139349

[38] Gallo V Motley K Kemp SPT Mian S Patel T James L. Concussion and long-term cognitive impairment among professional or elite sport-persons: a systematic review. J Neurol Neurosurg Psychiatry. (2020) 91:455–68. 10.1136/jnnp-2019-321170 32107272 PMC7231435

[39] Kim JJ Gean AD. Imaging for the diagnosis and management of traumatic brain injury. Neurotherapeutics. (2011) 8:39–53. 10.1007/s13311-010-0003-3 21274684 PMC3026928

[40] Paterakis K Karantanas AH Komnos A Volikas Z. Outcome of patients with diffuse axonal injury: the significance and prognostic value of MRI in the acute phase. J Trauma. (2000) 49:1071–5. 10.1097/00005373-200012000-00016 11130491

[41] Sapin V Gaulmin R Aubin R Walrand S Coste A Abbot M. Blood biomarkers of mild traumatic brain injury: State of art. Neurochirurgie. (2021) 67:249–54. 10.1016/j.neuchi.2021.01.001 33482234

[42] Thelin EP Zeiler FA Ercole A Mondello S Büki A Bellander BM. Serial sampling of serum protein biomarkers for monitoring human traumatic brain injury dynamics: a systematic review. Front Neurol. (2017) 8:300. 10.3389/fneur.2017.00300 28717351 PMC5494601

[43] Bazarian JJ Biberthaler P Welch RD Lewis LM Barzo P Bogner-Flatz V. Serum GFAP and UCH-L1 for prediction of absence of intracranial injuries on head CT (ALERT-TBI): a multicentre observational study. Lancet Neurol. (2018) 17:782–9. 10.1016/S1474-4422(18)30231-X 30054151

[44] Wong VS Langley B. Epigenetic changes following traumatic brain injury and their implications for outcome, recovery and therapy. Neurosci Lett. (2016) 625:26–33. 10.1016/j.neulet.2016.04.009 27155457 PMC4915732

[45] Di Pietro V Yakoub KM Scarpa U Di Pietro C Belli A. MicroRNA signature of traumatic brain injury: from the biomarker discovery to the point-of-care. Front Neurol. (2018) 9:429. 10.3389/fneur.2018.00429 29963002 PMC6010584

[46] Liberati A Altman DG Tetzlaff J Mulrow C Gøtzsche PC Ioannidis JP. The PRISMA statement for reporting systematic reviews and meta-analyses of studies that evaluate healthcare interventions: explanation and elaboration. BMJ. (2009) 339:b2700. 10.1136/bmj.b2700 19622552 PMC2714672

[47] Tricco AC Lillie E Zarin W O'Brien KK Colquhoun H Levac D. PRISMA extension for scoping reviews (PRISMA-ScR): checklist and explanation. Ann Intern Med. (2018) 169:467–73. 10.7326/M18-0850 30178033

[48] Kennedy CE Fonner VA Armstrong KA Denison JA Yeh PT O'Reilly KR. The Evidence Project risk of bias tool: assessing study rigor for both randomized and non-randomized intervention studies. Syst Rev. (2019) 8:3. 10.1186/s13643-018-0925-0 30606262 PMC6317181

[49] Hooijmans CR Rovers MM de Vries RB Leenaars M Ritskes-Hoitinga M Langendam MW. SYRCLE's risk of bias tool for animal studies. BMC Med Res Methodol. (2014) 14:43. 10.1186/1471-2288-14-43 24667063 PMC4230647

[50] Wyczechowska D Harch PG Mullenix S Fannin ES Chiappinelli BB Jeansonne D. Serum microRNAs associated with concussion in football players. Front Neurol. (2023) 14:1155479. 10.3389/fneur.2023.1155479 37144000 PMC10151480

[51] Svingos AM Asken BM Bauer RM DeKosky ST Hromas GA Jaffee MS. Exploratory study of sport-related concussion effects on peripheral micro-RNA expression. Brain Inj. (2019) 33:1–7. 10.1080/02699052.2019.1573379 30704304 PMC7412735

[52] Ma SQ Xu XX He ZZ Li XH Luo JM. Dynamic changes in peripheral blood-targeted miRNA expression profiles in patients with severe traumatic brain injury at high altitude. Mil Med Res. (2019) 6:12. 10.1186/s40779-019-0203-z 31036067 PMC6489315

[53] Mitra B Major B Reyes J Surendran N Bain J Giesler LP. MicroRNA biomarkers on day of injury among patients with post concussive symptoms at 28-days: a prospective cohort study. Microrna. (2024) 13:233–9. 10.2174/0122115366297817240613065052 38982917

[54] Petrova TA Kondratyev SA Kostareva AA Rutkovskiy RV Savvina IA Kondratyeva EA. miR-21, miR-93, miR-191, miR-let-7b, and miR-499 expression level in plasma and cerebrospinal fluid in patients with prolonged disorders of consciousness. Neurol Int. (2022) 15:40–54. 10.3390/neurolint15010004 36648968 PMC9844494

[55] Seršić LV Alić VK Biberić M Zrna S Jagoić T Tarčuković J. Real-time PCR quantification of 87 miRNAs from cerebrospinal fluid: miRNA dynamics and association with extracellular vesicles after severe traumatic brain injury. Int J Mol Sci. (2023) 24:4751. 10.3390/ijms24054751 36902179 PMC10003046

[56] Di Pietro V Porto E Ragusa M Barbagallo C Davies D Forcione M. Salivary MicroRNAs: diagnostic markers of mild traumatic brain injury in contact-sport. Front Mol Neurosci. (2018) 11:290. 10.3389/fnmol.2018.00290 30177873 PMC6109773

[57] Di Pietro V O'Halloran P Watson CN Begum G Acharjee A Yakoub KM. Unique diagnostic signatures of concussion in the saliva of male athletes: the Study of Concussion in Rugby Union through MicroRNAs (SCRUM). Br J Sports Med. (2021) 55:1395–404. 10.1136/bjsports-2020-103274 33757972 PMC8639909

[58] Johnson JJ Loeffert AC Stokes J Olympia RP Bramley H Hicks SD. Association of salivary MicroRNA changes with prolonged concussion symptoms. JAMA Pediatr. (2018) 172:65–73. 10.1001/jamapediatrics.2017.3884 29159407 PMC5833519

[59] Lv J Zeng Y Qian Y Dong J Zhang Z Zhang J. MicroRNA let-7c-5p improves neurological outcomes in a murine model of traumatic brain injury by suppressing neuroinflammation and regulating microglial activation. Brain Res. (2018) 1685:91–104. 10.1016/j.brainres.2018.01.032 29408500

[60] Miao W Bao TH Han JH Yin M Yan Y Wang WW. Voluntary exercise prior to traumatic brain injury alters miRNA expression in the injured mouse cerebral cortex. Braz J Med Biol Res. (2015) 48:433–9. 10.1590/1414-431x20144012 25760028 PMC4445667

[61] He XC Wang J Du HZ Liu CM Teng ZQ. Intranasal administration of agomir-let-7i improves cognitive function in mice with traumatic brain injury. Cells. (2022) 11:1348. 10.3390/cells11081348 35456028 PMC9027059

[62] Johnson D Cartagena CM Tortella FC Dave JR Schmid KE Boutté AM. Acute and subacute microRNA dysregulation is associated with cytokine responses in the rodent model of penetrating ballistic-like brain injury. J Trauma Acute Care Surg. (2017) 83:S145–S9. 10.1097/TA.0000000000001475 28452880

[63] Balakathiresan N Bhomia M Chandran R Chavko M McCarron RM Maheshwari RK. MicroRNA let-7i is a promising serum biomarker for blast-induced traumatic brain injury. J Neurotrauma. (2012) 29:1379–87. 10.1089/neu.2011.2146 22352906 PMC3335133

[64] Sajja VSSS Jablonska A Haughey N Bulte JWM Stevens RD Long JB. Sphingolipids and microRNA changes in blood following blast traumatic brain injury: an exploratory study. J Neurotrauma. (2018) 35:353–61. 10.1089/neu.2017.5009 29020847

[65] Qin R Zhou J Chen C Xu T Yan Y Ma Y. LIN28 is involved in glioma carcinogenesis and predicts outcomes of glioblastoma multiforme patients. PLoS ONE. (2014) 9:e86446. 10.1371/journal.pone.0086446 24475120 PMC3901701

[66] Cai WY Wei TZ Luo QC Wu QW Liu QF Yang M. The Wnt-β-catenin pathway represses let-7 microRNA expression through transactivation of Lin28 to augment breast cancer stem cell expansion. J Cell Sci. (2013) 126:2877–89. 10.1242/jcs.123810 23613467

[67] Heneghan HM Miller N Kelly R Newell J Kerin MJ. Systemic miRNA-195 differentiates breast cancer from other malignancies and is a potential biomarker for detecting noninvasive and early stage disease. Oncologist. (2010) 15:673–82. 10.1634/theoncologist.2010-0103 20576643 PMC3228012

[68] Liu WJ Xu Q Sun LP Dong QG He CY Yuan Y. Expression of serum let-7c, let-7i, and let-7f microRNA with its target gene, pepsinogen C, in gastric cancer and precancerous disease. Tumour Biol. (2015) 36:3337–43. 10.1007/s13277-014-2967-9 25549793

[69] Donat CK Scott G Gentleman SM Sastre M. Microglial activation in traumatic brain injury. Front Aging Neurosci. (2017) 9:208. 10.3389/fnagi.2017.00208 28701948 PMC5487478

[70] Loane DJ Kumar A. Microglia in the TBI brain: The good, the bad, and the dysregulated. Exp Neurol. (2016) 275:316–27. 10.1016/j.expneurol.2015.08.018 26342753 PMC4689601

[71] Turtzo LC Lescher J Janes L Dean DD Budde MD Frank JA. Macrophagic and microglial responses after focal traumatic brain injury in the female rat. J Neuroinflammation. (2014) 11:82. 10.1186/1742-2094-11-82 24761998 PMC4022366

[72] Yao X Liu S Ding W Yue P Jiang Q Zhao M. TLR4 signal ablation attenuated neurological deficits by regulating microglial M1/M2 phenotype after traumatic brain injury in mice. J Neuroimmunol. (2017) 310:38–45. 10.1016/j.jneuroim.2017.06.006 28778443

[73] Essandoh K Li Y Huo J Fan GC. MiRNA-mediated macrophage polarization and its potential role in the regulation of inflammatory response. Shock. (2016) 46:122–31. 10.1097/SHK.0000000000000604 26954942 PMC4949115

[74] Banerjee S Xie N Cui H Tan Z Yang S Icyuz M. MicroRNA let-7c regulates macrophage polarization. J Immunol. (2013) 190:6542–9. 10.4049/jimmunol.1202496 23667114 PMC3679284

[75] Wang X Wang HX Li YL Zhang CC Zhou CY Wang L. MicroRNA Let-7i negatively regulates cardiac inflammation and fibrosis. Hypertension. (2015) 66:776–85. 10.1161/HYPERTENSIONAHA.115.05548 26259595

[76] Chin AC PERK-STING. Signaling drives neuroinflammation in traumatic brain injury. J Neurosci. (2020) 40:2384–6. 10.1523/JNEUROSCI.2881-19.2020 32188742 PMC7083527

[77] Abdullah A Zhang M Frugier T Bedoui S Taylor JM Crack PJ. STING-mediated type-I interferons contribute to the neuroinflammatory process and detrimental effects following traumatic brain injury. J Neuroinflammation. (2018) 15:323. 10.1186/s12974-018-1354-7 30463579 PMC6247615

[78] Lazzarino G Amorini AM Signoretti S Musumeci G Caruso G Pastore FS. Pyruvate dehydrogenase and tricarboxylic acid cycle enzymes are sensitive targets of traumatic brain injury induced metabolic derangement. Int J Mol Sci. (2019) 20:5774. 10.3390/ijms20225774 31744143 PMC6888669

[79] Di Pietro V Amorini AM Tavazzi B Vagnozzi R Logan A Lazzarino G. The molecular mechanisms affecting N-acetylaspartate homeostasis following experimental graded traumatic brain injury. Mol Med. (2014) 20:147–57. 10.2119/molmed.2013.00153 24515258 PMC3966992

[80] Hopkins BL Nadler M Skoko JJ Bertomeu T Pelosi A Shafaei PM. A Peroxidase Peroxiredoxin 1-Specific Redox Regulation of the Novel FOXO3 microRNA Target let-7. Antioxid Redox Signal. (2018) 28:62–77. 10.1089/ars.2016.6871 28398822 PMC5695745

[81] Chen PY Qin L Barnes C Charisse K Yi T Zhang X. FGF regulates TGF-β signaling and endothelial-to-mesenchymal transition via control of let-7 miRNA expression. Cell Rep. (2012) 2:1684–96. 10.1016/j.celrep.2012.10.021 23200853 PMC3534912

[82] Ma Y Shen N Wicha MS Luo M. The Roles of the Let-7 Family of MicroRNAs in the regulation of cancer stemness. Cells. (2021) 10:2415. 10.3390/cells10092415 34572067 PMC8469079

[83] Mayr C Hemann MT Bartel DP. Disrupting the pairing between let-7 and Hmga2 enhances oncogenic transformation. Science. (2007) 315:1576–9. 10.1126/science.1137999 17322030 PMC2556962

[84] Sampson VB Rong NH Han J Yang Q Aris V Soteropoulos P. MicroRNA let-7a down-regulates MYC and reverts MYC-induced growth in Burkitt lymphoma cells. Cancer Res. (2007) 67:9762–70. 10.1158/0008-5472.CAN-07-2462 17942906

[85] Kumar MS Erkeland SJ Pester RE Chen CY Ebert MS Sharp PA. Suppression of non-small cell lung tumor development by the let-7 microRNA family. Proc Natl Acad Sci U S A. (2008) 105:3903–8. 10.1073/pnas.0712321105 18308936 PMC2268826

[86] Yu CC Chen YW Chiou GY Tsai LL Huang PI Chang CY. MicroRNA let-7a represses chemoresistance and tumourigenicity in head and neck cancer via stem-like properties ablation. Oral Oncol. (2011) 47:202–10. 10.1016/j.oraloncology.2010.12.001 21292542

[87] Yang X Cai H Liang Y Chen L Wang X Si R. Inhibition of c-Myc by let-7b mimic reverses mutidrug resistance in gastric cancer cells. Oncol Rep. (2015) 33:1723–30. 10.3892/or.2015.3757 25633261

[88] Mao XG Hütt-Cabezas M Orr BA Weingart M Taylor I Rajan AK. LIN28A facilitates the transformation of human neural stem cells and promotes glioblastoma tumorigenesis through a pro-invasive genetic program. Oncotarget. (2013) 4:1050–64. 10.18632/oncotarget.1131 23846349 PMC3759665

[89] Tonini T Rossi F Claudio PP. Molecular basis of angiogenesis and cancer. Oncogene. (2003) 22:6549–56. 10.1038/sj.onc.1206816 14528279

[90] Liao YC Wang YS Guo YC Lin WL Chang MH Juo SH. Let-7g improves multiple endothelial functions through targeting transforming growth factor-beta and SIRT-1 signaling. J Am Coll Cardiol. (2014) 63:1685–94. 10.1016/j.jacc.2013.09.069 24291274

[91] Yuan H Zhang H Hong L Zhao H Wang J Li H. MicroRNA let-7c-5p suppressed lipopolysaccharide-induced dental pulp inflammation by inhibiting dentin matrix protein-1-mediated nuclear factor kappa B (NF-κB) pathway *in vitro* and *in vivo*. Med Sci Monit. (2018) 24:6656–65. 10.12659/MSM.909093 30238933 PMC6162970

[92] Jickling GC Ander BP Shroff N Orantia M Stamova B Dykstra-Aiello C. Leukocyte response is regulated by microRNA let7i in patients with acute ischemic stroke. Neurology. (2016) 87:2198–205. 10.1212/WNL.0000000000003354 27784773 PMC5123554

[93] Buonfiglioli A Efe IE Guneykaya D Ivanov A Huang Y Orlowski E. let-7 MicroRNAs regulate microglial function and suppress glioma growth through toll-like receptor 7. Cell Rep. (2019) 29:3460–71.e7. 10.1016/j.celrep.2019.11.029 31825829

[94] Coleman LG Zou J Crews FT. Microglial-derived miRNA let-7 and HMGB1 contribute to ethanol-induced neurotoxicity via TLR7. J Neuroinflammation. (2017) 14:22. 10.1186/s12974-017-0799-4 28118842 PMC5264311

[95] Rom S Dykstra H Zuluaga-Ramirez V Reichenbach NL Persidsky Y. miR-98 and let-7g^*^ protect the blood-brain barrier under neuroinflammatory conditions. J Cereb Blood Flow Metab. (2015) 35:1957–65. 10.1038/jcbfm.2015.154 26126865 PMC4671116

[96] Schulte LN Eulalio A Mollenkopf HJ Reinhardt R Vogel J. Analysis of the host microRNA response to Salmonella uncovers the control of major cytokines by the let-7 family. EMBO J. (2011) 30:1977–89. 10.1038/emboj.2011.94 21468030 PMC3098495

[97] Teng GG Wang WH Dai Y Wang SJ Chu YX Li J. Let-7b is involved in the inflammation and immune responses associated with Helicobacter pylori infection by targeting Toll-like receptor 4. PLoS ONE. (2013) 8:e56709. 10.1371/journal.pone.0056709 23437218 PMC3577724

[98] Bernstein DL Rom S. Let-7g^*^ and miR-98 reduce stroke-induced production of proinflammatory cytokines in mouse brain. Front Cell Dev Biol. (2020) 8:632. 10.3389/fcell.2020.00632 32766248 PMC7379105

[99] Guan H Fan D Mrelashvili D Hao H Singh NP Singh UP. MicroRNA let-7e is associated with the pathogenesis of experimental autoimmune encephalomyelitis. Eur J Immunol. (2013) 43:104–14. 10.1002/eji.201242702 23079871 PMC3650085

[100] Lepp AC Carlone RL. MicroRNA dysregulation in response to RARβ2 inhibition reveals a negative feedback loop between MicroRNAs 1, 133a, and RARβ2 during tail and spinal cord regeneration in the adult newt. Dev Dyn. (2015) 244:1519–37. 10.1002/dvdy.24342 26332998

[101] Gandhi R Healy B Gholipour T Egorova S Musallam A Hussain MS. Circulating microRNAs as biomarkers for disease staging in multiple sclerosis. Ann Neurol. (2013) 73:729–40. 10.1002/ana.23880 23494648

[102] Zhuang Y Peng H Mastej V Chen W. MicroRNA regulation of endothelial junction proteins and clinical consequence. Mediators Inflamm. (2016) 2016:5078627. 10.1155/2016/5078627 27999452 PMC5143735

[103] Bernstein DL Zuluaga-Ramirez V Gajghate S Reichenbach NL Polyak B Persidsky Y. miR-98 reduces endothelial dysfunction by protecting blood-brain barrier (BBB) and improves neurological outcomes in mouse ischemia/reperfusion stroke model. J Cereb Blood Flow Metab. (2020) 40:1953–65. 10.1177/0271678X19882264 31601141 PMC7786850

[104] Zhang J. Dongwei Zhou Zhang Z Xinhui Qu Kunwang Bao Guohui Lu. miR-let-7a suppresses α-Synuclein-induced microglia inflammation through targeting STAT3 in Parkinson's disease. Biochem Biophys Res Commun. (2019) 519:740–6. 10.1016/j.bbrc.2019.08.140 31547989

